# Management and outcomes of blunt and penetrating traumatic aortic injuries in children

**DOI:** 10.1016/j.jvscit.2026.102230

**Published:** 2026-03-12

**Authors:** Priscilla Tanamal, Sophia Trinh, Claudie Sheahan, Amit Chawla, Amanda Tullos, Marie Unruh, Malachi Sheahan

**Affiliations:** a Department of Surgery, Louisiana State University Health Sciences Center, New Orleans, LA; b Division of Vascular Surgery, Louisiana State University Health Sciences Center, New Orleans, LA; c Division of Vascular Surgery, Geisinger Health Systems, Danville, PA

**Keywords:** Aortic, Pediatric, Trauma, Vascular

## Abstract

Our study reviewed the management and outcomes of pediatric aortic injuries at our institution. Patients ≤18 years old with traumatic aortic injuries (2010-2023) were identified after retrospective review of a Level 1 trauma center registry. Of 16 patients (mean age, 13.3 years), the majority suffered blunt mechanism (62.5%) and were managed by endovascular repair, open repair, resuscitative thoracotomy, or anticoagulation alone. Penetrating injuries underwent endovascular repair or attempted open repair via exploratory laparotomy and/or resuscitative thoracotomy. Mortalities occurred <24 hours from presentation. Operative and endovascular repair had no complications at follow-up and was associated with excellent long-term outcomes.

Trauma is the most common cause of mortality in the pediatric population.^1^ Despite this, significant vascular injury is rare, occurring in only 0.6% of pediatric trauma patients.^2^ Although thoracic and abdominal aortic injury consists of a small subset of these injuries,^3^ they are often fatal, with survival rates as low as 65% of those who make it to the hospital.^4^ The current literature consists of evaluation of management using the National Trauma Data Bank (NTDB)^5^^,^^6^ and case reports and case series regarding the presentation, management, and outcome of aortic injury in children.^7^, ^8^, ^9^, ^10^ Studies demonstrate the importance of early diagnosis based on mechanism of injury and appropriate imaging,^11^^,^^12^ consistent with guidelines for the adult population.^13^ Historically, operative management has been focused on primary repair of the aorta over interposition graft or endovascular aortic stents.^14^ Recently, stent grafts have become more widely used.^5^^,^^7^^,^^13^^,^^14^ In the adult population, an endovascular approach has been found to have decreased mortality compared with open repair.^15^ Although studies have shown a trend towards endovascular repair for these injuries and good outcomes for this patient population,^5^^,^^6^^,^^16^^,^^17^ no strong evidence-based guidelines exist regarding the management of aortic injuries specific to the pediatric population. Our study aims to review the treatment and outcomes of pediatric aortic injuries at our institution.

## Methods

A retrospective review was performed of a prospectively maintained Level 1 trauma center registry. Approval for the study was obtained from the Louisiana State University Health Sciences Center Institutional Review Board. Between 2010 and 2023, patients ≤18 years of age with traumatic aortic injuries were included. Demographics, vitals upon presentation, injury grade and location, management, outcome variables, concurrent injuries, and follow-up data were gathered. The Glasgow Coma Scale (GCS) was measured based on eye opening (1-4), verbal (1-5), and motor (1-6) responses for a total score of 3 to 15. The Injury Severity Score (ISS) was calculated using the sum of the squares of the highest Abbreviate Injury Scale scores from the three most severely injured body regions. Descriptive statistics were performed.

## Results

Between 2010 and 2023, 16 patients aged 18 years and younger were identified with traumatic aortic injuries. Eleven were male (68.7%), and five were female (31.3%). The mean age was 13.3 years (range, 3-18 years). Mean systolic blood pressure and heart rate at presentation were 103.6 ± 19.0 mmHg and 119.2 ± 25.9 beats per minute, respectively. GCS was 9.9 ± 5 at presentation, and ISS was 28.1 ± 7.4. Mechanism was blunt in 10 cases (62.5%) and penetrating in six (37.5%). Nine of the blunt injuries were from car accidents, and one was from a fall. All penetrating injuries were due to firearms. Details of the aortic injury including anatomical site and grade of blunt injury and other nonaortic injuries are listed in Table I.

**Table I:** Aortic injury characteristics and other injuries

MOI	Age, years	Specific MOI	Anatomical site of injury	Grade of injury	Other injuries	Complications/outcomes
Blunt	3	MVC	Descending aorta	III	Cardiac contusion, tricuspid valve injury, sternal fracture	Sternal wound dehiscence following tricuspid valve repair
	6	MVC	Infrarenal aorta	IV	Small bowel injury	
	6	MVC	Aortic bifurcation	III	None	
	8	MVC	Infrarenal aorta	III	Renal laceration, spine fracture	
	11	MVC	Aortic isthmus	III	Splenic laceration, pneumothorax, femur fracture, rib fracture	Wound infection
	14	MVC	Infrarenal aorta	III	Sternal fracture, thoracic spine fracture, pulmonary contusion	
	15	MVC	Infrarenal aorta	II	Small bowel injury	
	16	MVC	Aortic isthmus	III	Pneumothorax, rib fracture, splenic laceration, humerus fracture	Dissection from REBOA placement
	16	Fall	Descending aorta	IV		Death
	17	MVC	Descending aorta	III	Pneumothorax, pelvic fractures, rib fractures, splenic hematoma, liver laceration	
Penetrating	15	GSW	Visceral aorta		IMV, SMV, colon	Death
	16	GSW	Descending aorta		Lung laceration	Death
	16	GSW	Descending aorta		Diaphragm, stomach, colon injury; liver laceration	Abdominal wound dehiscence, pleural effusion
	17	GSW	Visceral aorta		Stomach and renal injuries	Death
	18	GSW	Visceral aorta		Liver laceration	Death
	18	GSW	Descending aorta		Spine fracture, rib fractures	MI, pleural effusion

*GSW*, Gunshot wound; *IMV*, inferior mesenteric artery; *MI*, myocardial infarction; *MOI*, mechanism of injury; *MVC*, motor vehicle crash; *REBOA*, resuscitative endovascular balloon occlusion of the aorta; *SMV*, superior mesenteric artery.

Management of the blunt injuries consisted of endovascular repair (n = 5), open repair (n = 3), resuscitative thoracotomy (n = 1), and anticoagulation alone (n = 1). Management of the penetrating injuries consisted of endovascular repair (n = 2) or attempted open repair (exploratory laparotomy, resuscitative thoracotomy, or both) (n = 4). All interventions were performed within 3 days from presentation. Type and length of stent graft, access site, sheath size, and method of access closure for endovascular interventions as well as specifics of open repair are outlined in Table II.

**Table II:** Management and outcomes of aortic injuries

MOI	Age, years	Specific MOI	Anatomical site of injury	Grade of injury	Management of aortic injury	Access Right CFA Left CFA	Closure Right CFA Left CFA	Outcome	Follow-up, months
Blunt	3	MVC	Descending aorta	III	TEVAR with 10 × 22 mm NuMED Covered CP stent graft	4 Fr 12 Fr	Manual pressure Manual pressure		65.0
	6	MVC	Infrarenal aorta	IV	Open repair with 10-mm Dacron tube graft				61.0
	6	MVC	Aortic bifurcation	III	Open repair with 10-mm aorto-iliac and 8-mm limb to contra iliac				102.5
	8	MVC	Infrarenal aorta	III	Open repair with 10-mm Dacron tube graft				54.5
	11	MVC	Aortic isthmus	III	TEVAR with 21 × 100 mm GORE→ TAG→ stent graft	5 Fr 18 Fr	Manual pressure Patch angioplasty		4.0
	14	MVC	Infrarenal aorta	III	EVAR with 16 × 14.5 × 70 mm GORE→ Excluder→ iliac limb stent graft	12 Fr 5 Fr	Perclose Proglide ×2 Mynx		3.0
	15	MVC	Infrarenal aorta	II	Anticoagulation				15.5
	16	MVC	Aortic isthmus	III	TEVAR with 21 × 100 mm GORE→ TAG→ stent graft	7 Fr (REBOA) 18 Fr	Patch angioplasty Patch angioplasty		27.7
	16	Fall	Descending aorta	IV	Thoracotomy			Death	
	17	MVC	Descending aorta	III	TEVAR with 21 × 100 mm GORE→ TAG→ stent graft	– 18 Fr	– Perclose Proglide ×2		85.1
Penetrating	15	GSW	Visceral aorta		Thoracotomy + exploratory laparotomy			Death	
	16	GSW	Descending aorta		Thoracotomy			Death	
	16	GSW	Descending aorta		TEVAR with 21 × 100 mm GORE→ TAG→ stent graft	5 Fr 18 Fr	Primary repair Primary repair		14.7
	17	GSW	Visceral aorta		Exploratory laparotomy			Death	
	18	GSW	Visceral aorta		Thoracotomy + exploratory laparotomy			Death	
	18	GSW	Descending aorta		TEVAR with 23 × 30 mm GORE→ Excluder→ cuff stent graft	5 Fr 16 Fr	Mynx Patch angioplasty		52.8

*CFA*, Common Femoral Artery; *EVAR*, endovascular aortic repair; *Fr*, French; *GSW*, gunshot wound; *MOI*, mechanism of injury; *MVC*, motor vehicle crash; *REBOA*, resuscitative endovascular balloon occlusion of the aorta; *TEVAR*, thoracic endovascular aortic repair.

Five of the injuries resulted in death. One mortality resulted from a blunt injury (1/10; 10%), and four from a penetrating injury (4/6; 66.7%). All patients who died had received an exploratory laparotomy and/or resuscitative thoracotomy during attempts to control their aortic injury. All mortality occurred within less than 24 hours of presentation. The patients who did not survive their injury, regardless of whether it was blunt or penetrating, presented either having received cardiopulmonary resuscitation (CPR) in the field or immediately upon arrival to the emergency department. The death following a fall occurred after the patient received CPR in the emergency room followed by a clamshell thoracotomy revealing hemothorax, hemopericardium, and peri-aortic hematoma extending up the descending thoracic aorta into the arch with active extravasation and cardiac arrest. Deaths following penetrating injury included three patients who underwent thoracotomy in the emergency department; two of those achieved return of spontaneous circulation and were taken to the operating room. While in the operating room, during their exploratory laparotomies, the patients again became pulseless with inability to achieve return of spontaneous circulation. Of the nine patients with blunt aortic injury who survived the first 24 hours, all but one had concomitant injuries. Four of the nine had pneumothorax or pulmonary contusion (44.4%), three had spine fractures (33.3%), three had splenic injury (33.3%), two had small bowel injury (22.2%), and two had sternal fractures (22.2%) (Table I). All patients with penetrating injury presented with concomitant injuries, including injuries to the colon, visceral vessels, lung, diaphragm, stomach, liver, kidney, spine, and ribs (Table I).

There were no complications related to aortic repair in either group identified throughout the follow-up. Patients did have complications secondary to interventions for concomitant nonaortic injuries such as sternal wound dehiscence following tricuspid valve repair, femoral artery dissection following resuscitative endovascular balloon occlusion of the aorta placement in the trauma bay, abdominal wound dehiscence, pleural effusions, and myocardial infarction (Table I). Average follow-up was 45.4 months, with a median of 52.8 months. Five patients had surveillance imaging documented in the electronic medical record, which included ultrasound, computed tomography (CT), or chest x-ray.

## Discussion

Our study demonstrates a 31.3% mortality rate in this pediatric aortic injury cohort that made it to our Level 1 trauma center. All deaths occurred less than 24 hours from presentation, and all patients presented already undergoing or requiring immediate initiation of CPR upon presentation. For those patients who survived the initial aortic repair, whether open or endovascular, survival was noted to be excellent with few long-term complications.^3^^,^^18^ Although delayed repair has also been shown to be safe in select patients,^18^ none of our patients underwent intervention more than 3 days after injury. In our cohort, only one patient received medical management alone. The patient with a presented with a Grade II blunt aortic injury—imaging showed an aortic filling defect secondary to an intimal tear with large intramural thrombus, which was managed with anticoagulation alone to manage the risk of embolization and aortic occlusion. Subsequent serial imaging demonstrated resolution of the Grade II blunt aortic injury. Although nonoperative management was the outlier in our study, NTDB data showed that 67.3% of patients did not receive any surgical intervention.^6^

Blunt injury continues to be the most common cause of trauma in pediatric patients, and the predominant mechanism of pediatric aortic injury is motor vehicle-related.^4^ However, it is important to note the increasing trend of pediatric fatalities due to firearm injuries, which surpassed motor vehicle-related injuries in 2019.^15^ Four of six of our patients with penetrating aortic trauma succumbed to their injuries within 24 hours. With this increasing prevalence, it is essential to continue to study and report the management and outcomes for both penetrating and blunt aortic injuries in children.

Although open aortic repair has been the historical standard for pediatric trauma patients, endovascular repair has become more frequently used.^7^^,^^9^^,^^17^^,^^19^^,^^20^ Consistent with our cohort, recent NTDB data also showed that endovascular repair was the most common surgical intervention.^6^ Endovascular repair has been found to have the same initial outcomes as open repair^21^ and should be considered in those with anatomically suitable injuries. One study examining the treatment of thoracic aortic injury demonstrated a lower risk of in-hospital mortality with thoracic endovascular aortic stent (TEVAR) compared with nonoperative management and open aortic repair.^5^ When stratified by age, nonoperative management may be safe for younger children, whereas endovascular repair may improve outcomes for the adolescent age group, especially in those with Grade 1 to 2 blunt aortic injuries.^22^ Zambetti et al showed no difference in morbidity or mortality when comparing young children (ages 1-9 years) against adolescents (ages 10-19 years) suffering blunt aortic injury despite significantly more adolescents undergoing TEVAR than the younger cohort. It did show, however, that adolescents who underwent TEVAR had significantly lower mortality than adolescents who did not. Studies have demonstrated the safety and benefit of balloon expandable covered stents—beneficial in the smaller pediatric vessel due to their smaller delivery profile and the ability for repeat dilation.^23^^,^^24^

The Society for Vascular Surgery clinical practice guidelines published in 2010 recommend TEVAR as the treatment for traumatic thoracic aortic injury based on improved outcomes compared with open aortic repair, for anatomically suitable injuries. For the pediatric population, options are more limited as endovascular devices are not produced in sizes and designs appropriate for all pediatric patients. In our series, the only patients with penetrating aortic injuries that survived had undergone an endovascular repair of a thoracic aortic injury, likely representing a subset of patients stable enough to be deemed candidates for transfer to the hybrid operating room.

Additional considerations exist for the pediatric patient for long-term surveillance after endovascular repair. In the adult population, endovascular repair of aortic injury at the index event has been shown to prevent the progression of late aortic-related complications.^16^ This requires a long period of time for surveillance with CT imaging. The risks and benefits of radiation exposure in children must be carefully considered. Arbuthnot et al examined the risks and benefits of routine chest CT use and the risk of radiation-induced malignancy and found that the risk for cancer was greater than the positive finding of thoracic aortic injury from blunt trauma.^25^ Improved understanding of the management and short- and long-term outcomes of these injuries will also aid in developing more standardized surveillance. Mehl et al described a case in which a child was initially managed nonoperatively due to a lack of visible injury to the major mediastinal vessels but eventually developed a descending aortic pseudoaneurysm identified on a surveillance scan 10 days after the injury. This was then managed via endovascular stent graft. Kim et al found progression of aortic injuries between 48 hours and 30 months,^18^ demonstrating the need for continued surveillance.

Although this dataset does not account for the pediatric population who succumb to their aortic injuries prior to hospital admission, there is still a need to understand outcomes of those that do. NTDB studies have allowed for the examination of these injuries, interventions, and outcomes^5^^,^^6^; however, without the granularity of the specific interventions. Pooling the pediatric aortic injury experience from other Level 1 trauma centers would allow for a more comprehensive understanding of these patients at presentation and as they grow. In a survey of pediatric surgeons and pediatric trauma medical directors, vascular injuries were more likely to be operatively managed by vascular surgeons, except for emergent subclavian injuries.^26^ The excellent outcomes following the repair of aortic injuries in children who survive the initial presentation highlight the importance of vascular surgery coverage in all accredited pediatric trauma centers.

## Conclusions

Pediatric traumatic aortic injury is rare but can be associated with high mortality, especially in the setting of a penetrating mechanism. Patients who survive past their initial presentation and undergo operative or endovascular repair have excellent long-term outcomes.

## Funding

None.

## Disclosures

None
